# Girls of New York

**Published:** 1857-12

**Authors:** 


### HALL'S JOURNAL OF HEALTH.

OUR LEGITIMATE SCOPE IS ALMOST BOUNDLESS: FOR WHATEVER BEGETS PLEASURABLE AND HARMLESS FEELINGS, PROMOTES HEALTH; AND WHATEVER INDUCES DISAGREEABLE SENSATIONS, ENGENDERS DISEASE.

VOL. IV.] DECEMBER, 1857. [NO. XII.

WE aim to show how disease may be avoided, and that it is best, when sickness comes, to take no medicine without consulting an educated physician.

### THE GIRLS OF NEW YORK.

WHAT will become of them this long and dreary winter, when the pinching times have thrown thousands of them out of employment? Many of them will pine away in want and honorable destitution, and quietly pass into the friendly grave, where toil is no more exacted, and where the busy fingers are at rest forever!

Not long ago, a young seamstress came to us, *as a favor*, at a dollar a day, besides her board, beginning the day's work at half past eight o'clock! and yet, there are multitudes of females among us who could sew just as well, and would most willingly do it, at a quarter the price, if it could be done without its being known to any but themselves; and other multitudes are there, who would quite as willingly act as cooks and chambermaids for a dollar a week, "*if it was not for the name of the thing.*"

There are thousands of families, in New York and around it, who would be but too glad to board and give a dollar a week to girls who would cook, or sew, or wash, or sweep. Now, can not some plan be devised to bring these classes together, to the mutual satisfaction and joy of both?—accommodating the families, and saving the unemployed from hunger and desperation, and even crime.

There are thousands of cooks and chambermaids, in this and other large cities, as to whom, two dollars a week—*one* dollar a week—is out of all proportion to their merit in such times as these; while there are other thousands, more intelligent, more tidy, more capable, who would be happy to serve for half a dollar a week, plentiful meals, and comfortable sleeping apartments being secured to them.

